# Extra-Renal Manifestations of Complement-Mediated Thrombotic Microangiopathies

**DOI:** 10.3389/fped.2014.00097

**Published:** 2014-09-08

**Authors:** Johannes Hofer, Alejandra Rosales, Caroline Fischer, Thomas Giner

**Affiliations:** ^1^Department of Pediatrics I, Innsbruck Medical University, Innsbruck, Austria

**Keywords:** TMA, aHUS, complement, extra-renal TMA, neurovascular complications, cardiovascular complication, gastrointestinal complications

## Abstract

Thrombotic microangiopathies (TMA) are rare but severe disorders, characterized by endothelial cell activation and thrombus formation leading to hemolytic anemia, thrombocytopenia, and organ failure. Complement over activation in combination with defects in its regulation is described in an increasing number of TMA and if primary for the disease denominated as atypical hemolytic-uremic syndrome. Although TMA predominantly affects the renal microvasculature, extra-renal manifestations are observed in 20% of patients including involvement of the central nerve system, cardiovascular system, lungs, skin, skeletal muscle, and gastrointestinal tract. Prompt diagnosis and treatment initiation are therefore crucial for the prognosis of disease acute phase and the long-term outcome. This review summarizes the available evidence on extra-renal TMA manifestations and discusses the role of acute and chronic complement activation by highlighting its complex interaction with inflammation, coagulation, and endothelial homeostasis.

## Background

Thrombotic microangiopathies (TMA) develop on the basis of microvascular endothelial cell injury with subsequent formation of platelet rich microthrombi triggering Coombs negative hemolytic anemia (except for pneumococcal hemolytic-uremic syndrome), non-immune thrombocytopenia, and organ failure (1, 2). The underlying histological lesion is characterized by prominent endothelial cell swelling and detachment with sub-endothelial debris, thickened arteriole and capillary walls, and thrombosed vessel lumina (1, 2).

Under normal conditions, intact endothelial surfaces are resistant to complement activation and are non-thrombogenic (3–5). The endothelial glycocalyx is the first barrier of the vascular wall and thus in direct contact to the vessel lumen and is crucial for endothelial integrity by acting as mechanosensor and permeability barrier in addition to its important role in anti-thrombotic and anti-inflammatory pathways (3, 4). It was shown that endothelial surface glcocalyx with its heparane sulfates is crucial for the regulatory potential of the complement regulator factor H (CFH) and of proteins involved in the coagulation cascade (3, 6–11). Thus, it is hypothesized that vascular glycocalyx heterogeneity could serve as a potential modifier of complement-mediated TMA (3).

Endothelial cells and their secretion products are tightly linked to the coagulation, fibrinolysis, and complement cascades. Prostacyclines, von-Willebrand-factor (vWF), thrombomodulin, nitric oxide, tissue plasminogen activator inhibitor, and further more lead to the loss of endothelial thromboresistance and widespread platelet aggregation creating a cycle of vasoconstriction and further thrombus formation (2, 3, 5).

The complement system forms an autonomous part of innate immunity and is essential for the mediation and concertation of inflammatory reactions, protection against invading organisms, and thus maintenance of tissue homeostasis (12, 13). The complement system consists of multiple proteins, acting in a cascade like manner to eliminate foreign invaders. During the activation process, inflammatory peptides are cleaved from inactive proteins (zymogenes) exerting multiple biological functions. Three activation pathways, the classical (CP), the lectin (LP), and the alternative pathway (AP) converge in the production of the “membrane attack complex” or “terminal complement complex” (TCC) (14–16). Binding of C1 (composed of a recognition unit, C1q, and a tetramer of the zymogens C1r and C1s) to an activator (mainly immunocomplexes) is the initial step leading to CP complement activation (14–16). Mannose-binding lectins (MBL) and ficolins, which are capable to bind on specific surfaces of microorganisms or dying cells, initiate complement activation via the LP (14–16). The AP is initiated spontaneously by unspecific C3 hydrolysis leading to a constant C3b production with covalent deposition onto plasma exposed surfaces. On foreign surfaces, e.g., bacterial surfaces, C3b triggers the activation of the AP. Without regulation, even small triggers can lead to a self-preserving amplification of the AP with consumption of the complement components and a harmful response against “self”-surfaces (14–16).

On the host cells, this cascade is controlled by membrane-anchored (membrane-cofactor protein (MCP), complement receptor 1, decay accelerating factor, CD55 and CD59), and fluid phase regulators (C1 inhibitor, C4 binding protein, CFH, complement factor I (CFI), CFH related proteins 1–5 (CFHR 1–5), clusterin, and vitronectin) (14–16). Foreign targets and injured cells that lack these regulators are attacked by complement, forming the TCC into the cells membrane leading to its lyses.

Complement-mediated TMA and especially atypical hemolytic-uremic syndrome (aHUS) are linked to uncontrolled activation of the AP (1, 2, 13):

Mutations in complement proteins (CFH, MCP, CFI, complement factor B (CFB), C3, or thrombomodulin) as well as antibodies against complement factor H (CFH-Ab) can be found in around 60–70% of patients with aHUS (1, 2). However, similar complement alterations were detected in ∼30% of patients with *de novo* post-renal-transplant HUS, 70% with recurrent HUS after kidney transplantation, and in one patient with severe TMA and antibody-mediated rejection (1, 2). Moreover, complement alterations and mutations are detected in 86% (18/21) of patients with pregnancy-associated HUS, in 43% (3/7) patients with cobalamin C deficiency, and in TMA associated with hematopoietic stem-cell transplantation (HSCT) (1, 2). Complement protein variants and mutations were also anecdotally reported in patients with TMA associated with ticlopidine, cisplatin, and carboplatin as well as malignant hypertension (MH) (1, 2). Thus, patients with underlying complement-regulatory defects have an increased propensity to develop TMA upon trigger events.

**Figure 1:**
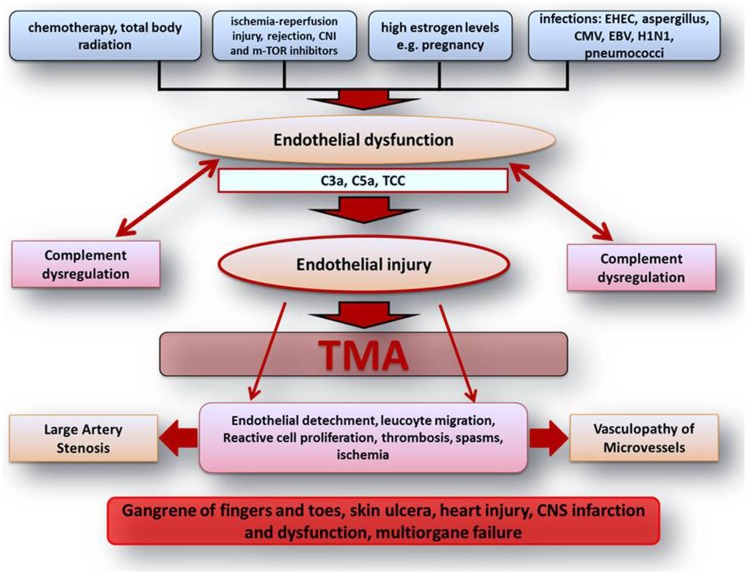
**Scheme on the pathophysiologic understanding of extra-renal complement-mediated TMA manifestations**. Endothelial dysfunction and injury represent the center of TMA pathogenesis. Different endothelial harming factors can lead to endothelial dysfunction. Especially on the basis of inherited or acquired defects of complement regulation and activation, those factors can lead to severe complement activation inducing widespread endothelial injury with the consequence of local and systemic activation of inflammation and coagulation. All these complex complement concerted and maintained processes may finally lead to vasculopathy of microvessels including vasa-vasorum with large artery stenosis and organ ischemia followed by multiple renal and extra-renal symptoms. C3a, complement component 3a; C5a, complement component 5a; CMV, cytomegalovirus; CNI, calcineurin inhibitor; CNS, central nerve system; EBV, Epstein–Barr virus; e.g., exempli gratia; EHEC, enterohemorrhagic *Escherichia coli*; H1N1, influence A virus subtype; m-TOR, mammalian target of rapamycin; TCC, terminal complement complex; TMA, thrombotic microangiopathy.

In general, the question why the glomerular endothelium is the main target of TMA is still unsolved. It is speculated that the specific fenestration of the glomerular endothelial cells leads to a higher susceptibility to complement activation and makes it more vulnerable to complement dysregulation as the glomerular basement membrane lacks surface bound complement regulators (2, 4, 12, 17). Moreover, the glomerular endothelial cell was shown to depend on vascular endothelial growth factor (VEGF) produced and secreted by podocytes allowing vascular endothelial regeneration and maintaining endothelial health (4, 18) (Figure 1). Thus, considering extra-renal TMA manifestations with endothelial dysfunction and injury in a more “protected” surrounding a higher cumulative endothelial stress potential may be needed to induce TMA as compared to the more vulnerable renal microvasculature. Considering those hypothesis, one may conclude that in addition to endothelial stress factors the level and quality of endothelial protective factors like nitric oxide production and VEGF secretion may modulate TMA pathogenesis and thus renal and extra-renal TMA manifestations.

A big player leading to endothelial stress is complement dysregulation and activation on cell surfaces, additionally triggered and stimulated by different infections and toxins (shigatoxin, neuraminidase) (4, 12, 17).

Although complement-mediated TMA predominantly affects the renal microvasculature, extra-renal TMA manifestations are observed in up to 20% of patients including involvement of the central nerve system (CNS), cardiovascular system, lungs, skin, skeletal muscle, and gastrointestinal tract (1, 2, 12, 19, 20). Those extra-renal complications may not only be observed during disease acute phase but can also manifest to some degree years after it (1, 2, 12, 19, 20) and are thought to result from a chronic over activation/dysregulation of the complement system, on the basis of an altered endothelial cell integrity with a predominance of anti-protective and potentially endothelial cell damaging factors (inflammation, drugs, hypertension, radiation, complement activation, etc.) (Figure 1) (2, 4, 12, 20).

In this review article, we discuss the role of acute and chronic complement activation and dysregulation with respect to the spectrum of extra-renal TMA complications.

## Cerebrovascular TMA Manifestations

Central nerve system involvement is the most frequent extra-renal organ manifestation of aHUS (10–48%) (1, 2, 20–24). Symptoms are heterogeneous including irritability, drowsiness, convulsions, encephalopathy, diplopia, cortical blindness, hemiparesis or hemiplegia, stupor, and coma (1, 2, 20–24). Thus, CNS involvement represents a major cause of mortality and morbidity in affected individuals.

When considering neurological involvement due to complement-mediated TMA, a differentiation to secondary CNS phenomena induced by electrolyte dysbalances, uremia, and arterial hypertension is essential. CNS complications due to arterial hypertension [reversible posterior leukoencephalopathy syndrome with posterior white matter hyper intensity (PRES)] may show similar clinical presentations including vision loss and seizures and both entities may be interrelated in complement-mediated TMA (1). Moreover, rare CNS manifestations due to steno-occlusive lesions of the carotid arteries and cerebral lesions due to microvascular TMA lesions have to be differentiated. Interestingly, vascular lesions of large arteries in patients with complement-mediated TMA (Table 1) are reported mainly in patients with ESRD on long-term dialysis. The involved arteries are mainly from the carotid and vertebral arteries leading to ischemic CNS manifestations.

**Table 1:** **Overview of case reports describing cerebrovascular CNS manifestations (CNS, TMA, and macrovascular CNS manifestations)**.

Reference	Age onset/sex	Defect	Characteristics	Imaging/biopsy results	Specific therapy	Outcome
**CNS TMA**						
(25)	19 months/f	*CFH*-Polym	aHUS onset with fluctuating level of consciousness, prolonged, generalized, tonic–clonic seizures, hemiparesis; EEG showing diffuse slow-wave encephalopathy; In addition cardiovascular instability with poorly contractile left ventricle and a LV-EF of 30%	MRI: subtle bilateral parenchymal abnormalities	Eculizumab started within 12 h after diagnosis	Subtle weakness in right-thumb and finger otherwise age adequate neurologic investigations
(26)	4 years/m	*CFH/CFHR1*	Despite intense PT ESRD 8 months after onset; 1 month thereafter bilateral nephrectomy due to uncontrolled hypertension; 3 days after nephrectomy sudden onset of partial complex epileptic seizures without laboratory signs of aHUS recurrence and with normal blood pressure; 1 month thereafter recurrence of generalized seizures; 8 months later third episode of seizures with decreased hemoglobin, low haptoglobine, and moderate thrombocytopenia; persistent deep confusion and violent agitation over 2 days	First MRI (at onset of CNS symptoms): normal; Second MRI: normal; Third MRI: bilateral symmetrical hyperintensities on cerebral pedunculas, caudate nuclei, putamens, thalami, hippocampi, and insulae; fourth MRI (after initiation of PT): completely normal	After third episode of CNS symptoms with MRI lesions suggestive for TMA start with intense PE and after 4 weeks PE replaced by PI	Normal clinical examination except for high blood pressure
(27)	50 years/f	n/a	During first disease flair (day three) unresponsive seizures; EEG: diffuse cerebral dysfunction	Initial CT: unremarkable, MRI 28 days after Eculizumab start: subacute right parietal lobe infarction with areas of hemorrhagic conversion; suspected tiny subacute infarct within the right cenrtum semiovale	Eculizumab started at day 6 after disease onset	Complete neurologic (and renal) recovery
(28)	11 years/f	*CFH*-Polym, *CFI*-Mut.	Partial response to PI and PE; multiple tonic–clonic seizures despite ongoing PE therapy; 2 days after first seizures vision loss and severe confusion; laboratory investigations showed hemolysis and thrombocytopenia	First MRI: bilateral, symmetrical hyperintensities on the occipital and posterior parietal lobes and edema; second MRI (2 months therafter): normal	Prior to CNS manifestation PT; thereafter eculizumab in addition to levetiracetam	Complete neurologic recovery after 1 month; normalization of MRI
	6 years/f	MCP-Mut.	Initially response to PE and HD with a clear hematologic response after 5 days; on day six patient developed tonic–clonic seizures, followed by vision loss and nystagmus – in parallel worsening hemolysis and thrombocytopenia	MRI: bilateral, hyperintense white matter lesions in the occipital and posterior parietal regions	Eculizumab was initiated after MRI	Five weeks after first eculizumab dose complete neurologic, hematologic, and renal recovery
(29)	25 years/m	*De novo* TA-TMA	Ktx at 12 years of age due to FSGS; uneventful course till age of 25 years; at 25 years acute onset of left-sided weakness preceded by general fatigue and progressive forgetfulness in the previous 2 months. Lab at time of admission showed mild thrombopenia, with low normal hemoglobin, and LDH	First MRI/A: right-sided temporo-parietal and thalamic lesions; missing flow signal of the right middle cerebral artery; second MRI/A: additional ischemic lesions of the left-sided thalamus and both occipital regions; Brain biopsy: extensive necrosis and arteriolar hyalinosis; Post-mortem examination: disseminated TMA of brain, lungs, and renal allograft	Cyclosporin A, prednisolone, valsartan, and darbepoetin alpha at time of CNS manifestation; no specific TMA therapy	After first MRI/A the patient was discharges with ASS; 4 weeks later admission due to listlessness and mutism; 5 days thereafter bilateral blindness was discovered and Cyclosporin A was paused; second MRI/A was performed; development of severe arterial hypertension, rapidly declining platelets and slightly increased LDH; C3/C4 and CH50 normal, no further complement analyses
**MACROVASCULAR CNS MANIFESTATIONS**						
(30)	20 months/f	Fam.	At diagnosis development of gangrenous lesions of finger and toes with thromozytopenia, increased LDH, fragmentocytes, and creatinine; 90 days after aHUS onset seizures and acute hemiparesis	First MRI/A: ischemia of the right frontoparietal region and steno-occlusive lesions of both internal carotid arteries and patent vertebral arteries; no laboratory signs of TMA	After aHUS onset 16 PE sessions and four PI leading to aHUS recurrence	n/a
(31)	4 years/f	Stx-HUS	Stx-HUS onset with ESRD and need for PD. 3 months after onset various neurologic complaints including tonic movements of the left limbs, transient vision loss, and intermittent dysarthria	MRI/A: acute infarction in the right middle cerebral artery territory with bilateral occlusions of the distal internal carotid arteries with multiple small collaterals similarly to moyamoya disease; C3, C4, CH50, and CFH concentration normal, no further complement studies performed	PD; revascularization surgery;	Planed for renal transplantation; after revascularization persistent mild hemiparesis
(32, 33)	3 years/f	CFH-mut; fam.	Acute onset of aHUS proceeding to ESRD – first Ktx with aHUS recurrence and subsequent graft loss on day 3; nephrectomy was performed; with 12 years second Ktx under prophylactic pre and post-transplant PE. Eight weeks later creatinine increase and intense TMA lesions on biopsy, despite intensive PE graft loss and patient on HD. With 15 years episodic sensory and motor symptoms – first MRI; with 17 years third Ktx under prophylactic pre and post-transplant PE; postoperatively infarction in the right frontoparietal region with left-sided hemiparesis, focal seizures, and impaired consciousness. Thereafter progressive neurologic recovery without additional events under PE. Later on PE was switched to Eculizumab	First MRI/A: severe stenosis in proximal segments of the middle and anterior cerebral arteries; Further imaging: infarcts in the right frontal and frontoparietal regions	Prophylactic PE and later on due to allergic reactions Eculizumab	Progressive recovery of neurologic symptoms under PE
(34)	1 month/f	CFB-mut.	aHUS with ESRD at 4 months; due to severe arterial hypertension bilateral nephrectomy at the age of 1 year. Ktx at the age of 19 months; 15 days thereafter hematologic recurrence, treated with PE and IVIG. With 6 years again HD dependent, nephrectomy of the graft; with 10 years episodes of bilateral hemiparesis and loss of consciousness. Imaging studies showed multiple and intense stenoocclusive vascular lesions cerebral, pulmonal cardial and peripheral	MRI/A: bilateral stenosis of intracranial carotid arteries and left subclavian and vertebral arteries; CT-Angiography and cardiac catheterization: stenosis of all branches of pulmonary arteries, moderate pulmonary arterial hypertension and stenosis of distal anterior interventricular, right and marginal coronary arteries, right humeral artery, celiac trunk and splenic artery	Initially HD and nephrectomy. Recurrence after 1^st^ transplant treated with IVIG and PE. After graft loss nephrectomy and again HD. After onset of cerebrovascular symptoms attempt of carotid siphon angioplasty	Attempt of carotid siphon angioplasty, complicated by dissection and massive infarction leading to death
(35)	2 month/m	C3-mut.	Ten months after recovery of aHUS fulminant recurrence with ESRD despite PE. After 4.5 years PD and poor blood pressure control sudden onset of convulsions involving the right hand and face, followed by hemiparesis and aphasia (at that time normal RR and normal lab); MRI/A revealed stenoocclusive lesions; symptoms resolved completely without specific therapy; 1 year thereafter Ktx under prophylactic eculizumab therapy. At day four seizures of the right hand and face and aphasia; death at day 9 after transplantation	MRI/A: stenosis of the left internal carotid artery and bilateral collaterals of the middle cerebral arteries; signs of subacute stroke in the territory of the left middle cerebral artery. CT after renal Tx: massive ischemic lesions in the region of the middle cerebral arteries	PE after first recurrence – ESRD – PD – transplantation under prophylactic eculizumab	Development of severe stroke on day 4 after Ktx leading do death within 5 days despite eculizumab therapy
(36)	17 months/f	CFB and CFI mut. in addition to CFH-polym	Despite intensive PE ESRD 3 month after aHUS onset; with 3 years Ktx under prophylactic post-transplant PE and PI and nephrectomy of native kidneys. Despite prophylactic PT three aHUS recurrences leading to malignant hypertension and generalized seizures with transient focal neurologic symptoms (normal CT); with 4 years transplant nephrectomy and HD, PT was terminated. Seven years later, despite normal laboratory parameters development of a transient ischemic attack with severe neurological symptoms including vomiting, headache, aphasia, ataxia, confusion and weakness in both arms; measurement of C3 and C3dg showed ongoing complement activation and eculizumab was initiated under the suspicion of complement-mediated TMA like vasculopathy; Ktx 11 months after the TIA episode	CT at onset of CNS symptoms: normal, MRI/A at second severs CNS manifestation: total occlusion of the right carotid artery and near-occlusion of the left carotid artery. Repeated imaging over a period of one year showed no progression of vascular occlusions under eculizumab therapy	Eculizumab was started after severe TIA episode	Repeated imaging over a period of one year showed no progression of vascular occlusions under eculizumab therapy. The patient was successfully re-transplanted under eculizumab

*aHUS, atypical hemolytic-uremic syndrome; CFB, complement factor B; CFH, complement factor H; CFHR, CFH related protein; CFI, complement factor I; CNS, central nerve system; CT, computer tomography; EEG, electroencephalogram; ESRD, end stage renal disease; f, female; fam., familial; FSGS, focal segmental glomerulosclerosis; HD, hemodialysis; IVIG, intravenous immunoglobulins; Ktx, kidney transplantation; LDH, lactate dehydrogenase; LV-EF, left ventricular ejection fraction; m, male; MCP, membrane-cofactor protein; MRI, magnetic resonance imaging; MRI/A, MRI angiography; Mut., mutation; n/a, not available; PD, peritoneal dialysis; PE, plasma exchange; PI, plasma infusion; Polym., polymorphism; PT, plasma therapy (PE and/or PI); Stx, shigatoxin, TIA, transient ischemic attack; TMA, thrombotic microangiopathy*.

The clinical presentation of cerebral TMA lesions and secondary CNS phenomena are often indistinguishable, thus brain magnetic resonance imaging (MRI) including MRI-angiography (MRI/A) is essential to differentiate CNS complications due to arterial hypertension (PRES) and those due to cerebral TMA and/or steno-occlusive lesions (1, 26, 34, 37).

The following MRI findings are described frequently in the literature during the acute phase of HUS and are currently interpreted as directly TMA-associated lesions (1, 26, 34, 37): symmetrical and mainly bilateral basal ganglia, brainstem, and deep white matter lesions on diffusion weighted imaging.

There is a distinctive similarity of radiologic lesions found in patients with shigatoxin-HUS and those with aHUS (26, 37–39), leading to the hypothesis that those lesions are caused by a microangiopathic, thrombotic processes triggered by endothelial cell damage due to or in addition to complement dysregulation, which is not only a key feature in aHUS but also in shigatoxin-HUS (40, 41).

Interestingly, even in patients with severe CNS involvement on acute imaging, progression, and clinical development was favorable for basal ganglia lesions (in those studies no differentiation between shigatoxin-HUS and aHUS was/could be made) (38, 39). In contrast, detection of hemorrhagic CNS lesions is associated with at least minor neurological dysfunction on the long-run or delayed neurological recovery (38, 39). In the case of recurrent aHUS, MRI findings can distinguish those TMA-associated CNS manifestations from secondary CNS involvement (e.g., hypertensive encephalopathy) with important implications on the further management of those patients (26).

The largest aHUS cohorts describe cerebrovascular complications due to aHUS onset in 5/45 (11%; 1 CFH, 1 CFI, 3 without known mutation) (24), 23/211 (11%; 5 CFH, 2 CFI, 1 C3, 1 CFH-Ab, 14 without known mutation) (23), 6/45 (13%; 1 C3, 1 CFB, 1 CFH-Ab, 3 without known mutation) (21), and 11/23 (48%; no genotyping) (22) patients. Cohort studies on patients with CFH-Ab HUS (42) describe CNS involvement in 56/138 (41%) (43), 8/32 (23.5%) (44), and 2/19 (11%) (45). In addition, there are a number of case reports on patients with distinctive TMA CNS manifestations (25–29, 46) and steno-occlusive vascular CNS manifestations (30–36) (Table 1). As reported by Koehl et al. (26), recurrence of aHUS can present as isolated CNS manifestation without laboratory signs of complement activation. Patients with intracerebral TMA manifestations responded to early therapy [plasma exchange (PE) and/or eculizumab] in a significant proportion, while patients with macrovascular involvement showed diverse outcomes from response to PE/eculizumab up to death despite intensive eculizumab treatment (Table 1). However, early therapeutic interventions seem to be essential for a positive outcome.

## Cardiovascular TMA Manifestations

Cardiovascular complications have been reported in about 10% of aHUS patients including cardiomyopathy, myocardial infarction, myocarditis, and heart failure as well as steno-occlusive coronary lesions (1, 20–24). Again, the differentiation between primary complement-mediated, TMA-associated defects and secondary phenomena due to volume overload and/or arterial hypertension is essential but not always clear.

Table 2 summarizes the available case reports on patients with complement-associated cardiovascular TMA manifestations (34, 47, 48). In addition to those detailed case reports, several cohort studies on aHUS describe cardiac manifestations:

**Table 2:** **Overview of case reports on cardiovascular TMA manifestations in aHUS**.

Reference	Age at onset/sex	Defect	Characteristics	Imaging/biopsy results	Specific therapy	Outcome
**CARDIAL TMA**						
(34)	1 month/f	CFB-mut.	Anephric patient (Ktx; aHUS recurrence, graft loss, nephrectomy) with sudden onset of neurovascular symptoms (further details see Table 1)	CT-angiography and cardiac catheterization: stenosis of all branches of pulmonary arteries, moderate pulmonary arterial hypertension, and stenosis of distal anterior interventricular, right and marginal coronary arteries, right humeral artery, celiac trunk, and splenic artery	After onset of cerebrovascular symptoms attempt of carotid siphon angioplasty	Attempt of carotid siphon angioplasty, complicated by dissection and massive infarction leading to death
(48)	1 year/f	CFH-mut.	PE and HD after aHUS onset, ongoing hemolysis and severe hypertension. Two months after disease onset with worsening proteinuria, arterial hypertension, renal insufficiency, anemia, thrombopenia, and dilated cardiomyopathy leading to cardiorespiratory arrest with subsequent resuscitation and mechanical ventilation; under ongoing PE the clinical condition slightly improved but cardiac dysfunction did not resolve; thus eculizumab was introduced	On echocardiography signs of cardiomyopathy; ejection fraction decreased (around 31%)	PE leading to slight improvement of cardiac function; after introduction of eculizumab normalization of cardiac function	Normalization of cardiac function
(47)	43 years/f	CFH-mut; fam	Onset of aHUS including nephrotic syndrome and pulmonary edema with low C3; kidney biopsy showed typical TMA lesions. PE led to hematologic recovery. On day 15 after beginning of PE the patient developed sudden circulatory arrest with pulse rates under 25/min and the patient died despite immediate resuscitation attempts	Cardiac ultrasound during circulatory arrest: pericardial effusion with tamponade; Necroscopy: myocardial infarction without obstruction of the coronary arteries; multiple microscopic cardiomyocyte necrosis were present; no coronary thrombi or atherosclerosis; the small vessels showed microscopic features of TMA; immunochemistry revealed activation of final pathway of complement	PE leading to hematology remission	Sudden cardio circulatory arrest at day 15 of PE followed by death

*aHUS, atypical hemolytic-uremic syndrome; CFB, complement factor B; CFH, complement factor H; CT, computer tomography; f, female; fam., familial; HD, hemodialysis; Ktx, kidney transplantation; m, male; MRI, magnetic resonance imaging; MRI/A, MRI angiography; Mut., mutation; n/a, not available; PD, peritoneal dialysis; PE, plasma exchange; PI, plasma infusion; PT, plasma therapy (PE and/or PI); TMA, thrombotic microangiopathy*.

Neuhaus et al. (22) report on heterogeneous clinical presentations of aHUS, describing 10 children with cardiomyopathy at discharge, two of them died, no genotyping was available. Venables et al. (49) describe eight patients with familial aHUS on the basis of a CFH/CFHR1 hybrid gene, one of those patients died of myocardial infarction 10 years after aHUS onset, another died of cardiac arrest 8 weeks after onset. In a cohort study on sporadic and familial aHUS, Noris et al. (23) investigated 273 aHUS patients (adults and children). Out of those, seven patients were documented as having cardiovascular disease during acute aHUS episodes, five of those with identified CFH mutations. In a cohort study of 45 patients with CFH-Ab-associated aHUS (44), 3 patients were described developing cardiac insufficiency on the long-run, one of them died. Analog, out of seven children with CFH-Ab-associated aHUS one died due to myocarditis (50). Roumenia et al. (51) report on 14 aHUS patients with C3 mutations, 7 patients had dilated cardiomyopathy, 5 during acute phase, 1 of those died at onset following a cardiovascular event.

In one case report (47), histological work up after necroscopy in a 43-year-old woman with CFH mutation caused aHUS and cardiac arrest was performed. The histology revealed TMA features of the small coronary vessels with intense TCC staining in the small vessels and infarcted cardiomyocytes.

Thus, especially patients with genetic or acquired CFH and C3 defects appear to be at highest risk for cardiac complications due to microangiopathic injury in the coronary microvasculature (20).

Interestingly cardiac involvement is described in a significant percentage of patients with thrombotic-thromboytopenic purpura (TTP) (52, 53), a disease entity characterized by “a disintegrin and metalloproteinase with a thrombospondin type 1 motif member 13” (ADAMTS13) protease deficiency related TMA. In autopsy studies, those patients presented evidence for cardiac TMA in 84% of the patients (53). In spite of these pathologic observations, cardiac symptoms and clinical cardiac abnormalities are rarely described in those patients. Although complement-mediated TMA represents a distinct disease entity than TTP, there are several similarities. The author of a systematic review on cardiac involvement in TTP concluded that the discrepancy between clinical and pathologic cardiac abnormalities may be due to under diagnoses of cardiac involvement, as the common symptoms of dyspnea and weakness may be primarily attributed to anemia and cardiac symptoms may also be overlooked because many patients are young and without cardiac risk factors (52). The same conclusions seem reasonable for complement-mediated TMA were only few studies have investigated cardiac involvement, although the limited data suggest cardiac involvement as an important cause of continuing mortality.

A study on the incidence and prognosis of acute heart failure in adult patients with TMA in general (54) found an acute heart failure prevalence of 9.5%. The incidence of acute heart failure did not differ between idiopathic and defined causes of TMA. Patients with heart failure experienced around twice the mortality of patients without heart failure despite receiving PE therapy. In surviving patients, left ventricular systolic function improved over time. Nevertheless, no complement studies were performed.

There are also a number of case reports on cardiac complications in shigatoxin-HUS (55–63) with outcomes varying from complete recovery, over stabile improvement till death (dilatative cardiomyopathy in five patients, myocardial infarction in one patient, myocarditis in two patients, tamponade in two patients).

**Figure 2:**
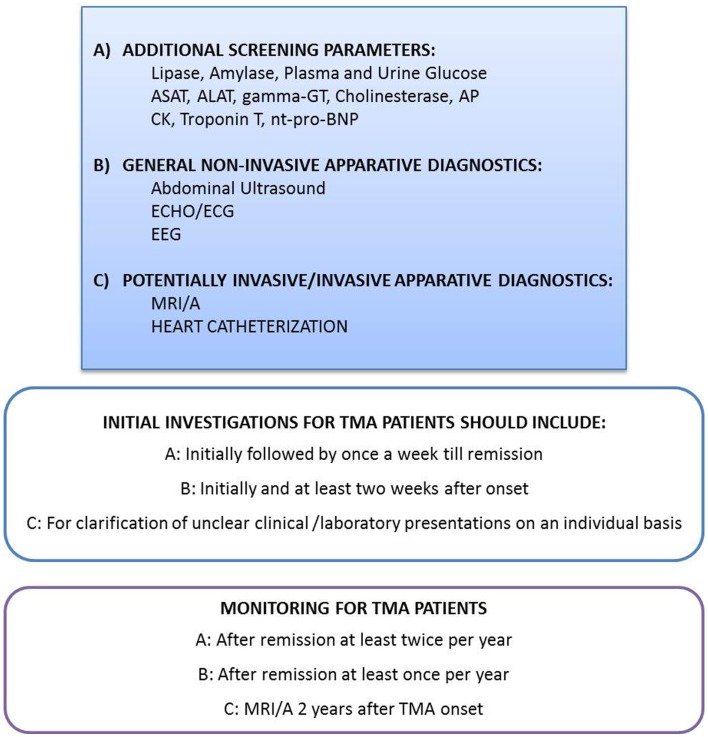
**Recommendation for evaluation and monitoring of extra-renal manifestations in patients with complement-mediated TMA**. Depending on the clinical presentation and possible symptoms, the physicians have to be aware of TMA associated, potentially devastating extra-renal TMA manifestations. A high level of suspicion may be needed to diagnose and treat those complications early in disease course. Thus, the initial laboratory and imaging investigations as well as the monitoring procedures have to reflect this knowledge. However, the indications for highly invasive diagnostic procedures like heart catheterization and MRI/A in small children with the need of total anesthesia have to be proven on an individual basis. The recommendation for MRI/A 2 years after onset in all patients with complement-mediated TMA excludes children with the need for total anesthesia, except those with clinical cerebrovascular symptoms. ALAT, Alanin-aminotransferase; AP, alkaline phosphatase; ASAT, aspartat-aminotransferase; CK, creatine kinase; ECG, electrocardiography; ECHO, echocardiography; EEG, electroencephalography; gamma-GT, gamma-glutamyl transpeptidase; MRI/A, magnetic resonance imaging/angiography; nt-pro-BNP, N-terminal pro brain natriuretic peptide.

Altogether, 7 aHUS patients with CFH mutations, 4 with CFH-Ab aHUS, 2 with a CFH/CFHR1 hybrid gene, 7 with C3 mutations, 1 with a CFB mutation, and 12 without a known mutation or without genotyping are described with cardiovascular events may be related to complement TMA. Thus, systemic screening for cardiac symptoms and manifestations are necessary and recommended on a regular basis for any patient with TMA, especially those with high risk mutations (Figure 2).

As detailed initially and in the discussion section, endothelial damage is at the center of TMA pathogenesis and cumulative endothelial harming factors may lead to smoldering TMA processes in several organs. Especially in the setting of renal and further organ transplantation on the basis of inherited or acquired complement-regulatory defects in combination with the complement activating potentials of the surgery and the further endothelial harming effects of several immunosuppressive drugs, especially calcineurin-inhibitors (CNI) and m-Tor inhibitors (Figure 1), extra-renal TMA, and thus cardiovascular manifestations earn increased attention. In a French study including 57 aHUS patients who had received a kidney transplant 7% died following cardiovascular events, two due to myocardial infarction, one due to acute cardiac failure, and one due to extensive stroke (64). As all patients were lacking major cardiovascular risk factors, endothelial lesions with TMA and the micro- and macrovasculature are the most plausible explanation (64).

Malignant hypertension is known to cause and/or trigger TMA (2). As detailed initially, complement-regulatory defects are found in a significant percentage of patients with MH and TMA. However, TMA may also cause MH, thus in patients with unexplained MH and any additional sign of TMA (hemolytic anemia, thrombocytopenia, renal failure, multiorgan damage) one has to consider a complement-mediated TMA process and complement screening at least on a functional basis is recommended to be performed.

The scarce data on therapy and outcome of cardiac and cardiovascular TMA do not allow any clear conclusions; nevertheless, as for cerebrovascular TMA late diagnosis and late initiation of PE and/or eculizumab seems detrimental for the further development.

## Peripheral Vascular and Skin TMA Manifestations

Gangrenous lesions of finger and toes are reported for only six children with aHUS in three independent case reports (30, 65, 66). In four out of six patients complement investigations were performed. Four showed decreased C3 levels. For only two of the six patients further genetic screening and antibody analyses are available, one showing a C3 mutation and the other one presenting with CFH-Ab:

A 4-year-old girl with CFH-Ab aHUS and decreased C3 levels developed gangrenous lesions of the fingers 2 days after aHUS onset. The patients unfortunately died 3 weeks later due to dialysis related complications (65).

Another child with a C3 mutation and decreased C3 levels showed aHUS onset at the age of 4 months. Five months later, she developed ischemic changes in fingers and toes progressing to gangrenous lesions despite intensive PE. Nevertheless, after initiation of eculizumab all left non-necrotic lesions regained perfusion wile aHUS activity subsided (65).

A 3-year-old boy presented with aHUS and decreased C3 levels. He was treated with fresh-frozen-plasma (FFP) infusions. As he developed ischemia of fingers and toes, PE was started but the ischemic lesions progressed to gangrenous and necrotic lesions, which had to be removed in the fourth month after disease onset (66).

Three further patients aged 20 months, 28 months, and 7 years at age of aHUS diagnosis developed gangrenous lesions of fingers. In one patient recovery of the lesions following PE was noticed. One patient showed additional carotid artery thrombosis after recovery from aHUS. The third patient died due to lack of appropriate therapy of uremia (30).

Notably, all patients reported with peripheral gangrenous lesions were children with severe forms of aHUS. Those reports lead to the suggestion that under certain conditions (including pro and anti-protective factors for endothelial cell function) TMA lesions can additionally affect small-medium arteries [luminal diameter of digital arteries about 100-fold larger than of the typically affected glomerular arteries (20)]. The low C3 levels and the further complement system anomalies (CFH-Ab, C3 mutation) further strengthen the hypothetic role of uncontrolled activation of the AP of complement in the pathogenesis of the described gangrenous lesions.

Skin involvement is a rare but maybe under diagnosed complication in the context of complement-mediated TMA and is reported for three patients in the literature. Ardiassino et al. (67) describe three cases of complement-mediated aHUS, two with a CFH mutation and one with CFH-Ab HUS, who developed skin lesions that completely recovered after establishing PT or eculizumab.

The macroscopic picture of all three patients with the clear diagnosis of aHUS was similar and compatible with a cutaneous small-vessel vasculopathy potentially induced by a complement-mediated TMA. In addition, the skin histology of the only patient who underwent biopsy was compatible with TMA. Moreover, there was a clear and impressive response to PT or eculizumab. None of the patients showed laboratory signs of aHUS activity. Thus, any skin lesion of unknown origin in patients with TMA should be considered as possible TMA manifestation despite laboratory signs of disease activity and may form an indication for a therapy with eculizumab or plasmatherapy [PT; PE and/or plasma infusion (PI)] (67).

## TMA and the Pulmonary System

Clinically, significant pulmonary TMA manifestations are only anecdotally reported in the literature and mainly in the context of multiorgan manifestations of severe and fulminant complement-mediated TMA. Approximately 5% of patients with aHUS present with a life-threatening multivisceral failure due to diffuse TMA including cardiac ischemic events, CNS complications, pancreatitis, intestinal bleeding, hepatic cytolysis, rhabdomyolysis, and pulmonary hemorrhage and failure. In those studies, post-mortem analysis revealed diffuse TMA lesions (1, 23, 24).

Abdallah et al. (68) report on a patient with acute renal failure and pulmonary hemorrhages. Kidney biopsy showed TMA and after prednisolone pulse and PE the patient recovered. ADAMTS13 activity and detailed complement analytics were not performed.

Losito et al. (69) report on a patient presenting with TMA of the kidneys and pulmonary hypertension. The patient showed normocytic anemia, a slightly raised LDH and a borderline C3 together with an increased creatinine and a nephritic syndrome. Pulmonary hypertension was diagnosed 3.5 years after TMA onset by echocardiography and heart catheterization. The patient complained of asthenia and exertional dyspnea as well as syncopes. At the same time, renal function worsened. Treatment with warfarin and epoprostenol was started, 18 months thereafter bosentan, an oral endothelin antagonist, was started and prostacyclin treatment discontinued. Under bosentan, the patient improved significantly including a stabilization and normalization of renal function. No further genetic analysis were performed. No further disease recurrences were documented. As endothelial dysfunction is one of the key players for TMA development, it is not surprising that endothelin with its vasoconstrictive potential has been advocated in TMA (70, 71). Interestingly treatment with bosentan in this patient led to improvement of both, renal function and pulmonary arterial hypertension. Eighteen years after initial presentation, this patient was diagnosed with cobalamin C deficiency, and treatment with hydroxocobalamin was instituted without significant changes on the clinical condition (72). Of note, pulmonary hypertension is observed in a significant proportion of patients with cobalamin C deficiency associated TMA (72). Although this entity does not present a primary complement-mediated TMA pathology, it shows that TMA processes within the pulmonary vasculature may lead to pulmonary hypertension and should be considered in patients with diagnosed TMA as possible underlying mechanisms for otherwise unexplained hypoxemic conditions.

Jodele et al. (73) report on five children after allogenic HSCT with pulmonary arterial hypertension and clinical signs of transplantation-associated thrombotic microangiopathy (TA-TMA) with kidney injury. Cyclosporine was discontinued in three of five patients and rituximab and PE showed no benefit for another patient. The histology in three of them showed global TMA in the pulmonary arterioles and cytomegalovirus (CMV) and adenovirus could be detected in the lung tissue of two patients. Interestingly, TA-TMA was found in 60% of patients with hypoxemic respiratory failure after HSCT. No detailed complement investigations were done for those patients. However, Jodele et al. (74) reported CFH-Ab in 3/12 patients undergoing HSCT. Antibodies were not detected in a control cohort consisting of 18 patients after HSCT with no evidence for TMA. Homozygous CFHR1 and three deletions were detected in three patients further documenting the importance of complement deregulation in HSCT TMA. In a recent guideline of HSCT TMA, TCC activation was one of the most important factors for prognosis of the disease (75). Moreover, the efficacy of eculizumab in several patients with HTCT TMA supports the importance of complement-mediated mechanisms for this TMA entity and thus for the role of complement-mediated TMA for pulmonary hypertension (75, 76).

## TMA and the Gastrointestinal Tract

Pancreatitis, intestinal bleeding and hepatic cytolysis are reported mainly in patients with life-threatening multivisceral failure due to diffuse TMA (5% of patients with aHUS) (1, 23, 24).

Diarrhea, especially bloody diarrhea as initial sign of *enterohemorrhagic Escherichia coli* enteritis/colitis is a “classical” presentation of shigatoxin-HUS (41). On the other hand, diarrhea as one important trigger factor for aHUS is nowadays well established (1, 21, 77). However, addressing the question of intestinal complement-mediated TMA involvement is highly difficult as only a small number of representative case reports are available.

A number of reports describe intestinal TMA in the context of post-solid-organ transplant TMA possibly triggered/caused by the use of calcineurin inhibitors (Cyclosporin A, Tacrolimus) (78, 79). Intestinal TMA was diagnosed histologically. Patients presented with abdominal colics, constipation, abdominal distension, strictures, occlusions, and even intestinal perforations (78–80). For those patients, no complement analyses are documented. The important role of complement activation for disease pathogenesis in these patients is questionable considering data of animal models provoking severe intestinal TMA induced by intraperitoneal tacrolimus donation only (81). However, no complement analyses were performed in those experiments as well.

Similarly to solid organ transplantations intestinal TMA events are reported in HSCT patients (80, 82). Again, no complement analyses are available and thus the role of complement activation for those events remains speculative.

Despite those reports, Ohanian et al. (27, 46) report on severe neurological and intestinal involvement in a patient with complement activation on the basis of aHUS responsive to eculizumab (genotyping is not reported). The 50-year-old female presented with diarrhea and abdominal pain subsequently developing fulminant pancolitis with acute renal failure and thrombocytopenia. Stool studies including EHEC were reported negative. Due to the fulminant pancolitis, the patients underwent total abdominal colectomy. The pathology confirmed ischemic colitis with features of TMA.

Pancreas involvement in patients with HUS or TTP is reported frequently (1, 21, 44, 83–85). Pancreatic ischemia caused by HUS/TTP may contribute to the common symptom of abdominal pain. The pathophysiology of development of pancreatitis is unknown, thus, TMA processes provoking intrapancreatic endothelial cell damage remain speculative explanations. Interestingly, it was hypothesized that pancreatitis may immediately precede an acute episode of HUS/TTP and may serve as trigger for TMA onset/recurrence (84, 85). It is suggested that the acute inflammatory response to pancreatitis, mediated by TNF-alpha, IL-1, IL-6, and IL-8 and further cytokines as well as the circulating pancreatic proteases may lead to endothelial activation, endothelial cell damage, activation of intravasal coagulation, and vWF-release and those mediating and aggravating TMA processes on the basis of potential predispositions (including complement-regulatory defects) (84, 85).

Pancreas involvement may lead to significant beta cell dysfunction with the development of insulin-dependent diabetes mellitus (IDDM) in rare cases even years after HUS onset (1, 83, 86). Geerdink et al. (21) reported on pancreas involvement at disease onset in 2/45 aHUS patients. One patient presented with a necrotizing pancreatitis and developed IDDM 10 years thereafter. It is speculated that a microangiopathic intrapancreatic process is responsible for tissue ischemia with the potential involvement of beta cells. Interestingly, beta cell dysfunction was reported for 3–15% of patients with shigatoxin-HUS (87). No complement analyses are available for these reports.

Transaminasemia at disease onset is reported for 13/27 (44) and 79/138 (43) patients with CFH-Ab HUS as well as 41/108 patients with HUS not further specified (43).

## TMA and the Sceletal Muscles

Rhabdomyolysis is reported in exceptional cases of TMA, mainly in the context of multiorgan failure (86, 88). It is suggested that rhabdomyolysis in those cases is caused by generalized muscle ischemia resulting from the microangiopathic process in muscle arterioles and capillaries (86). Reports on response to PE therapy and recurrence of TMA and rhabdomyolysis after weaning of PE support the causative role of the TMA process for the rhabdomyolysis (86, 88).

Micheletti et al. (89) report on a male patient with HUS onset at the age of 12 days. The initial HUS episode resolved without specific therapy and without sequelae. During follow-up, a steady increase in creatinine kinase (CK) and liver enzymes was observed, thus a standard protocol for evaluation of neuromuscular diseases was initiated without significant findings. At the age of 2 years following an acute febrile illness, the child developed a HUS recurrence with decreased C3 levels and rhabdomyolysis. After improvement of renal function and rhabdomyolysis on HD and regular FFP infusions, there were several episodes of hemolysis with an increase in CK over 2 months, thereafter full recovery of the HUS recurrence was achieved, but CK and liver enzymes remained above normal values. Complement and mitochondrial studies revealed CFH-gene risk polymorphisms on the one hand and a succinate coenzyme Q reductase (complex II) deficiency on the other hand. During the following 3 years on treatment with ubidecarenone, riboflavin, and carnitine, the child did not experience further HUS recurrences and no further rhabdomyolysis.

Pena et al. (86) report on a 28-month-old girl with acute onset of HUS and rhabdomyolysis, associated with pancreatic involvement and IDDM.

Andreoli et al. (88) explain rhabdomyolysis episodes in HUS patients as extra-renal TMA manifestations.

Saunier et al. (90) report on an infant with two episodes of rhabdomyolysis associated with HUS at 3 and 6 months of age. Investigations showed extremely reduced cytochrome *c* oxidase/succinate cytochrome *c* reductase ratio.

## Autoimmunity and TMA

Complement activation leading to TMA also occurs on the background of diverse autoimmune/connective tissue disorders (2). In systemic lupus erythematosus (SLE), the complement system is known to play an important role in the pathogenesis of disease, especially in the development of lupus nephritis (91). Immune complex mediated complement activation leads to C4d deposition on endothelial cells and might correlate with the development of TMA in lupus nephritis (92, 93). Not only the activation of the CP, but also the activation of LP and AP seem to play a role in the development of TMA (94). One study reports TMA lesions in 24% of 148 renal biopsies of Chinese lupus nephritis patients, many of them also presenting decreased CFH levels in plasma (95). Furthermore, variants of genes encoding CFHR1 and 3 may contribute to the development of SLE (96). Those homozygous deletions are related to CFH-Ab production in aHUS (42, 45), in addition these antibodies were detected in 6.7% of 60 Swedish SLE patients (97).

The antiphospholipid syndrome (APS) is characterized by arterial and venous thrombi and most frequently occurs in association with other autoimmune diseases, most commonly with SLE (in addition and independently of lupus nephritis) (98). Complement activation through antiphospholipid antibodies may also be involved in the development of TMA in the APS. This relationship has been shown in murine models (99), and one unique article reports on the success of eculizumab treatment in three patients with APS nephropathy (100).

In the antineutrophil cytoplasmatic autoantibodies (ANCA) – associated vasculitis (AAV), the endothelium is damaged through oxygen reactive species and proteolytic enzymes released by ANCA-bound neutrophils. In addition, neutrophils release properdin and CFB, which activate the AP that might lead to TMA. One recent review summarizes the reports on the coexistence of AAV and TMA (2).

Thrombotic microangiopathies also could play a role in the pathogenesis of further connective tissue diseases, especially in the renal involvement, such as systemic sclerodermia (101), Sjögrens Syndrome (102).

ANA positivity was reported in 7/27 patients with CFH-Ab HUS (Titer 1:80–1:1250; speckled pattern) (44).

A better knowledge about the role of complement and development of TMA in autoimmune and connective tissue diseases could serve as a rationale for the use of complement directed therapies in those disease entities. However, physicians treating patients with those disorders should be aware of possible TMA events and manifestations.

## Discussion

The excess in complement activation due to the inherited or acquired disorders of complement regulation is associated with significant thrombophilia (5, 103). Complement activation disrupts endothelial cell integrity and the physiologically thromboresistent endothelial phenotype, thus contributing to thrombotic occlusions in the micro- and even macro-vasculature (4, 5, 20, 103). Endothelial surface C3b deposition recruits white cells into the area of endothelial damage (3–5, 20, 103). In addition, stressed endothelial cells express P-selectines and further adhesion proteins leading to release of vWf and platelet aggregation (5, 20, 104). Complement-mediated damage of thrombocytes was shown to induce the expression of the inflammatory CD40 ligand, further amplifying a pro-inflammatory milieu at site of endothelial cell damage and thus the vasculature (104). The microangiopathic thrombotic lesions lead to mechanic hemolysis and free heme induces further C3b deposition with further promotion of C5a production and TCC insertion on endothelial cells (105). Interestingly, studies in paroxysmal–nocturnal–hemoglobinuria (PNH) and aHUS have shown that complement inhibition through eculizumab was able to significantly reduce markers of hemostatic activation (D-Dimer), endothelial activation [soluble vascular cell adhesion protein 1 (sVCAM-1)], and inflammation [soluble tumor-necrosis-factor 1 (sTNFR1)] (5).

As endothelial dysfunction due to multiple risk factors is a more random explanation for TMA pathogenesis, one has to question its interpretation on the basis of the above presented data. Extra-renal side effects are mainly reported for complement-mediated TMA forms; interestingly, in patients treated with VEGF-blockers, one study documented a high number of localized renal TMA events with or without hematologic signs of HUS (106), but no patients with further extra-renal manifestations strengthen our hypothesis that severe extra-renal TMA on the basis of endothelial dysfunction is additionally dependent on a complement-regulatory defect.

The presented cases lead to the assumption that despite the absence of overt disease recurrences and even in the setting of normal laboratory basic evaluations progression of extra-renal (and indeed renal) vascular lesions is possible and striking under certain circumstances, especially in the setting of high risk mutations in combination with long-term dialysis. Thus, it is the uncontrolled, subclinic, systemic complement activation, and dysregulation that cause inflammation and endothelial damage leading to renal and extra-renal manifestations on the basis of a thrombotic micro- and/or “macro”-angiopathy.

The important role of complement activation for vascular stenosis in general is documented in several studies, although the detailed mechanisms are still unclear (107, 108). Moreover, complement activation is present in human and experimental arteriosclerosis and TCC deposition correlates with disease state (107, 108). As already detailed above, complement activation leads to endothelial up regulation of adhesion molecules and release of leukotrienes and further cytokines, which concert leukocyte recruitment, activation, and transmigration together with the high potential chemotaxines C3a and C5a (4, 5, 20, 103). All these events cause vessel wall inflammation, further endothelial damage, vessel obstruction, and through release of platelet-derived-growth-factor (PDGF) vessel wall thickening and stenosis (4, 5, 20, 103). Of importance, most reported cases with vascular stenosis were on dialysis and hemodialysis filters were shown to induce complement activation. Thus, complement activation due to HD procedures could exacerbate in the setting of complement-regulatory defects and further contribute to advanced endothelial and vascular damage.

Reminiscing the different case summaries on extra-renal TMA manifestations detailed above, one can easily recognize the different levels of evidence concerning complement activation and deregulation for the presented patients. Especially for neurovascular, cardiovascular, and peripheral vascular and skin manifestations, the summarized evidence strongly supports a crucial role of complement activation and deregulation for the development of TMA and “macroangiopathies.”

Considering pulmonary and gastrointestinal involvement, the differentiation of directly complement-mediated TMA induced injury and secondary therapy and infection induced phenomena is more speculative, although clear evidence exists that complement triggered TMA can directly induce pulmonary hypertension and hemorrhage as well as distinctive gastrointestinal symptoms in some patients with mainly very severe TMA.

Physicians have to be aware of secondary TMA phenomena in patients with autoimmune disorders involving endothelial cells like SLE, APA syndrome, and AAV.

Of note, extra-renal complement-mediated TMA manifestations occur either in an acute setting at disease onset or under recurrences or as late manifestations of an continuous and smoldering complement activation process on the basis of regulatory defects in addition to potential TMA triggers like infections, CNI, radiation, etc. Especially, macrovascular lesions seem to progress even during symptom-free intervals, especially in patients with high risk mutations and ongoing HD.

These facts may stimulate the discussion on the prophylactic use of eculizumab to potentially prevent serious complement-mediated vascular damage especially for high risk patients on HD (CFH, CFB, CFI mutations, and CFH-Ab patients). Still the decision on how and when to use and wean eculizumab has to be decided on an individual basis considering renal and extra-renal perspectives in combination with the underlying pathophysiology of the affected patient.

The treating physician has to be aware of those possible extra-renal manifestations even despite disease acute phase and to initiate an appropriate monitoring of these patients (authors recommendation: see Figure 2). In general, the treating physician has to raise his awareness for extra-renal complement-mediated TMA processes with mild to severe manifestations in nearly all organ systems to be able to introduce individualized diagnostic work up and therapeutic interventions.

## Conflict of Interest Statement

Johannes Hofer, Alejandra Rosales, and Thomas Giner received honorarium from Alexion Pharmaceuticals Inc. and served on advisory boards. Caroline Fischer reports no conflict of interest.

## Abbreviations

AAV, antinuclear antiobody-associated vasculitis; ADAMTS13, a disintegrin and metalloproteinase with a thrombospondin type 1 motif member 13; aHUS, atypical HUS; ANCA, antinuclear antibodies; AP, alternative complement pathway; APS, anti phospholipid syndrome; CFH, complement factor H; CFH-Ab, complement factor H antibodies; CFHR, complement factor H related protein; CFI, complement factor I; CK, creatinine kinase; CMV, cytomegalovirus; CNI, calcineurin inhibitor; CNS, central nervous system; CP, classical complement pathway; FFP, resh frozen plasma; HD, hemodialysis; HSCT, hematopoietic stem-cell transplant; HUS, hemolytic-uremic syndrome; IDDM, insulin-dependent diabetes mellitus; LP, lectine complement pathway; MBL, mannose-binding lectine; MCP, membrane-cofactor protein; MRI, magnetic resonance imaging; MRI/A, MRI angiography; PDGF, platelet derived growth factor; PE, plasmaexchange; PI, plasma infusion; PRES, posterior reversible encephalopathy syndrome; SLE, systemic lupus erythematodes; sTNFR1, soluble tumor-necrosis-factor receptor 1; sVCAM-1, soluble vascular cell adhesion protein 1; TA-TMA, transplant-associated thrombotic microangiopathy; TCC, terminal complement complex; TMA, thrombotic microangiopathy; TTP, thrombotic thrombocytopenic purpura; VEGF, vascular endothelial growth factor; vWf, von-Willebrand-factor.
